# Diversification of the shell shape and size in Baikal Candonidae ostracods inferred from molecular phylogeny

**DOI:** 10.1038/s41598-023-30003-5

**Published:** 2023-02-20

**Authors:** Ivana Karanovic, Huyen T. M. Pham, Tatiana Sitnikova

**Affiliations:** 1 Department of Life Science, Research Institute for Convergence of Basic Science, College of Natural Sciences, Hanyang University, Seoul, 04763 Republic of Korea; 2 Department of Applied Chemistry and Biological Engineering, Graduate School, Department of Molecular Science and Technology, Ajou University, Suwon, 16499 Republic of Korea; 3 Limnological Institute, Siberian Branch, Russian Academy of Sciences, Irkutsk, Russia

**Keywords:** Evolution, Zoology

## Abstract

Ostracod shells are used extensively in paleontology, but we know little about their evolution, especially in ancient lakes. Lake Baikal (LB) is the world’s most important stronghold of Candonidae diversity. These crustaceans radiated here rapidly (12–5 Ma) and with an unprecedented morphological diversity. We reconstruct their molecular phylogeny with 46 species and two markers (18S and 16S rRNA), and use it to estimate the evolution of the shell shape and size with landmark-based geometric morphometrics (LBGM). High posterior probabilities support four major clades, which differ in node depth and morphospace clustering. After removing a significant allometry, the first three principal components (PCs) describe about 88% of total variability, suggesting a strong integration. Reconstructed ancestral shapes are similar for all four clades, indicating that diversification happened after colonization. Major evolutionary changes occurred from trapezoidal to elongated shapes. Sister species are separated in morphospace, by centroid size, or both, as well as by vertical and horizontal distributions in LB. Ostracod shell is a strongly integrated structure that exhibits high evolvability, with some extreme shapes, although mostly along the first PC. This is the first study that combines molecular phylogeny and LBGM for ostracods and for any LB group.

## Introduction

Evolvability of a morphological character describes a population potential to change in the direction of selection, and can be measured with LBGM^1,2^. A strong integration between or within structures is a good indicator of evolutionary directions^3,4^. Traits that are under strong selective pressure, usually those that perform multiple functions, generally display fewer changes over time^5^. Ostracod shell is a dorsally articulated bivalve shield, homologous to an overgrown head and thorax exoskeleton of other crustaceans, which provides protection for all other body parts, often serves as a brooding chamber, and aids mechanical stability in the environment^6^. Such versatile functions should result in limited changes during evolution.

Lake Baikal is a perfect place to study these phenomena, due to its age and an enormous diversity of ostracods^7^. It is the world’s deepest and by volume the largest freshwater rift lake^8^. Although the rifting probably started in the Late Cretaceous (~ 70 Ma), the lake formation is usually referred to the Oligocene (~ 30 Ma), with the deepest parts forming relatively recently (500–150 ka)^9,10^. It is divided into the south, central, and north basins, separated by the Selenga River delta and Academichesky Ridge, respectively. A dynamic geological and hydrological history set a stage for the evolution of a rich and mostly endemic fauna, with over 2500 species^11^. However, the true biodiversity of the lake is still insufficiently studied, especially from the abyssal zone^12^. Many animal groups have series of species flocks in LB, partly as a result of adaptive radiation^13,14^.

Ostracods in LB are represented by two major families. The family Cytherideidae number more than 60 species here^15^, all still in a single genus. Globally these are mostly marine animals, occasionally found in brackish habitats, with the exception of the LB group and several genera endemic to Lake Tanganyika^16^. The family Candonidae are exclusively freshwater globally, with diversity hotspots in LB^7^, Lake Ohrid^17^, and during the Late Miocene in Lake Pannon^18,19^. They number around 500 Recent species^20^, almost half of which live in LB and subterranean waters of Western Australia^21^.

**Figure 1:**
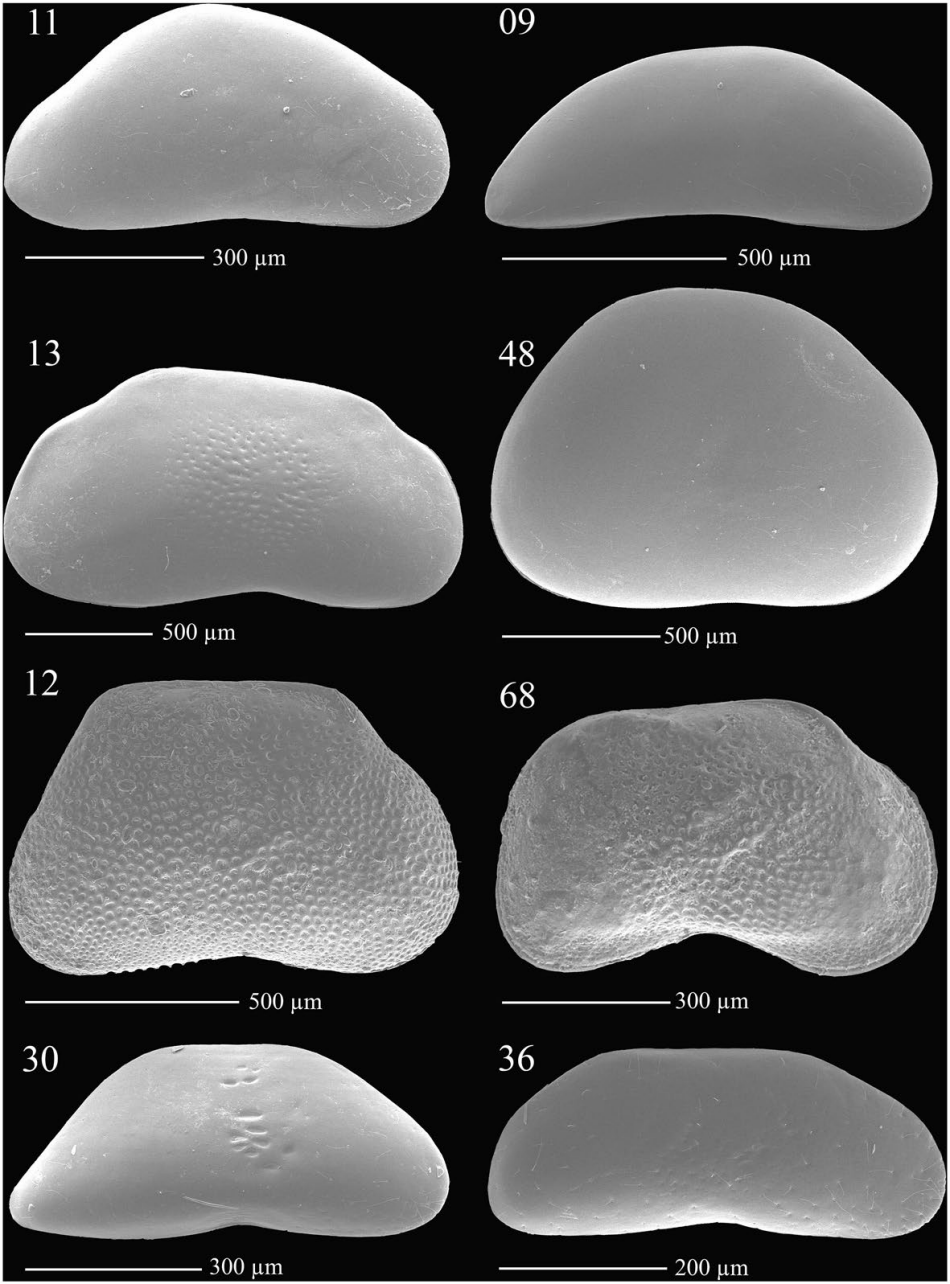
Examples of the shell shape diversity in Baikal Candonidae, representing all four major clades (**A**–**D**, see Fig. 3); numbers above scanning electron microscope photographs correspond to species codes in Supplementary Table S3, Supplementary Figs. S4, S5, and Figs. 3, 4, 5.

Candonidae from LB have been studied since the mid-twentieth century^22^, with a major taxonomic work accomplished by the early nineties and suggesting almost 100 endemics^23^. Most of them are insufficiently described according to modern taxonomic standards^7^, and they show an outstanding morphological diversity (Fig. 1). To properly address their phylogenetic position within the family, many researchers suggested a necessity of a revision^21,24^. Until now only two phylogenetic studies of Candonidae that contain representatives from LB have been published, one based on morphological characters^21^, the other on molecular markers^7^. Both demonstrated a polyphyletic nature of the four genera currently used. The molecular study^7^ included 10 species from LB and 28 species from around the world, and suggested at least two independent colonizations of the lake, with mayor radiation between 12 and 5 Ma.

The systematics of Candonidae globally is mostly based on the morphology of soft body parts, because their shells display extreme variability in shape, size, and ornamentation^20,25^. This poses problems for the taxonomy of fossil taxa (were only shells are preserved), and is reflected in frequent systematic changes and revisions, with little accomplishment^24,26^. Earlier studies^27^ showed that Candonidae have the highest diversity of shell shape among freshwater ostracod lineages, although this might be slightly exaggerated due to an old classification used. Quantitative analyses of the shell shape have sometime been applied in ostracodology to address problems of homoplasies^28,29^, correlation with ecological variables^30–32^, or cryptic species^33,34^. Although the use of morphometrics (both linear and geometric) in evolutionary studies has been constantly on the rise^35^, there are very few on ostracod shells, and only on the subgenus level and using morphology-based phylogeny^36,37^.

Despite the well-studied phylogenomic conflicts^38^, the phylogenetic assumptions derived from cladistic analyses of morphological traits gain more resolution with the addition of molecular data^39^. As a result of adaptive radiation, these conflicts often coincide with a fast morphological diversification^40^. Combining LBGM and phylogeny can stipulate adaptive radiation, with the convergent evolution as its main outcome^41,42^. This can be applied on a vast range of geological time scales^43,44^, and these methods can elucidate correlation between morphological and taxonomic diversity^45^.

**Figure 2:**
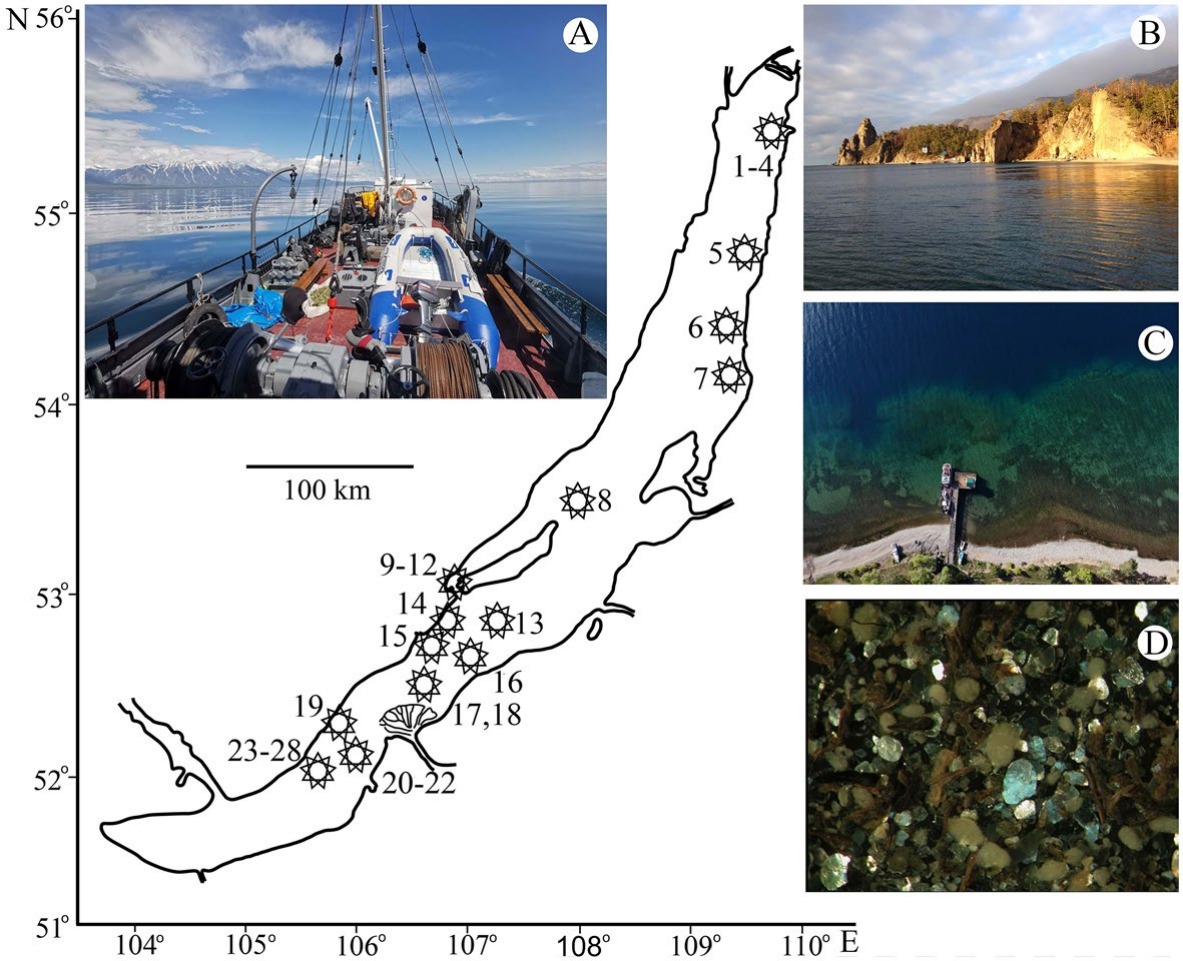
Map of Lake Baikal, with approximate positions of sampling localities (for details see Supplementary Note) and a selection of photographs from research expeditions: (**A**) Research Vessel “Titov” (photo: I. Khanaev); (**B**) Peschanaya Bay, Bolshiye Kolokonyi, close to locality no. 19 (photo: I. Karanovic); (**C**) Bolshiye Koty, close to localities nos. 27 and 28 (photo: I. Khanaev); (**D**) an example of bottom sediments at locality no. 28 (photo: T. Sitnikova).

Aims of our study were to reconstruct a molecular phylogeny of the LB Candonidae from all three basins (Fig. 2) and measure its contribution to the diversification of the shell shape and size. For this we used Procrustes coordinates of 48 landmarks (LMs) that best describe the overall outline of both valves. Because Candonidae display a significant size variation among species in most genera^20^, we tested the influence of evolutionary allometry on the shape variation. The results of our study could be applicable to a better understanding of the evolution of fossil ostracods, which could help their unstable systematics. Also, we hoped to get a better insight into the evolution of animals in ancient lakes.

## Results

**Figure 3:**
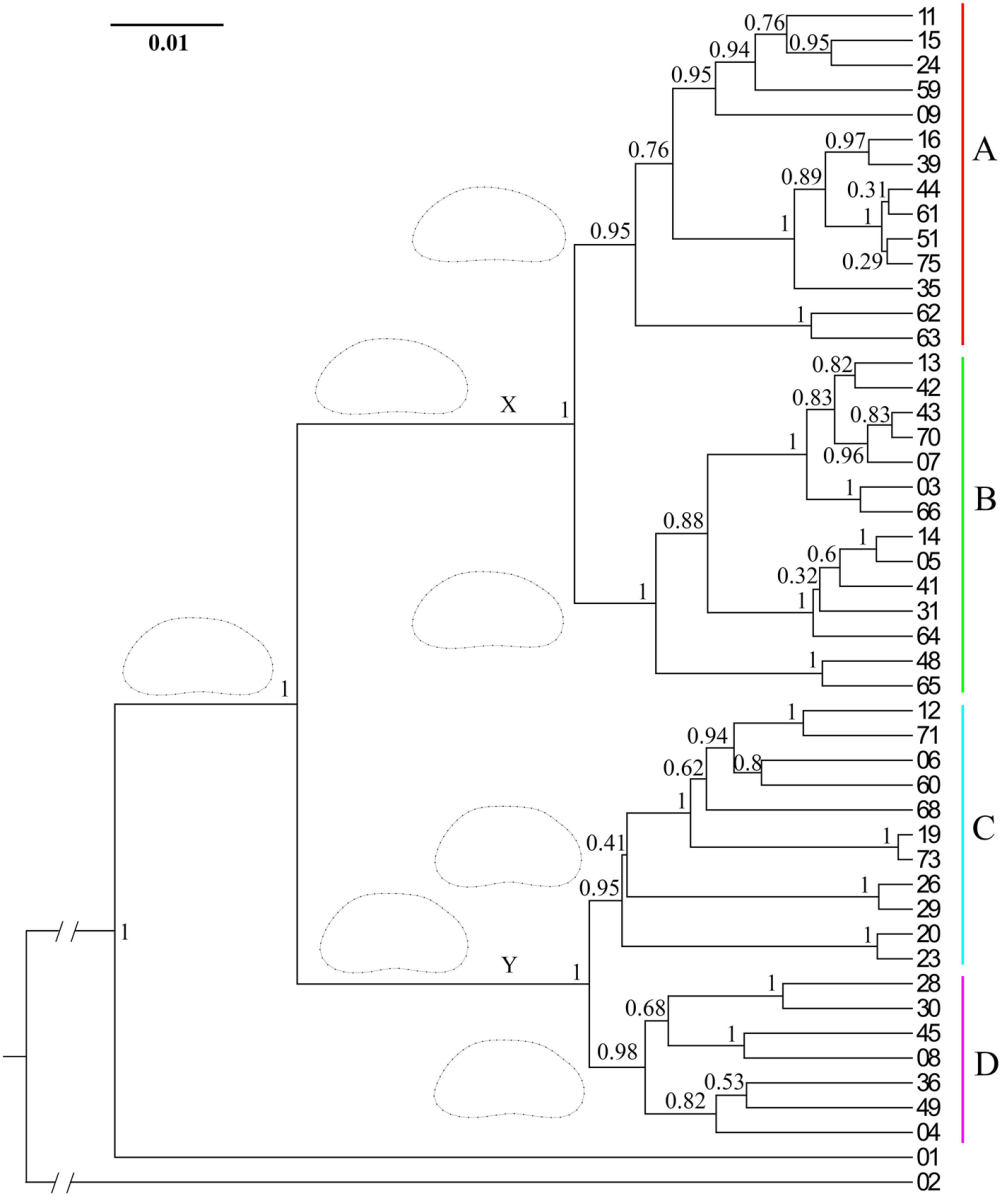
Bayesian Inference (BI) cladogram based on a concatenated alignment of partial 18S and 16S rRNA sequences from 46 Baikal Candonidae. X and Y, basal clades; A–D, major clades used for LBGM analyses (color codes correspond to PCA plots in Figs. 3 and 4 and in all Supplementary Figures). Numbers on branches show BI posterior probabilities. Terminal numbers are species codes (for details see Supplementary Table S3). Outlines represent ancestral shapes, reconstructed by plotting PCA scores onto the concatenated tree, using square-change parsimony.

Our concatenated DNA alignment was 1420 base pairs long, of which 926 were 18S, 441 were 16S, and the remaining 53 were binary codes of indels. The GTR + G + I was chosen as the best fit evolutionary model for the phylogenetic tree reconstruction, and the Tracer analyses of the BEAST results showed that the effective sample size for all measured parameters (posterior, likelihood, priors, tree likelihood, tree height, Yule model, birth rate, etc.) was far above the recommended 200, suggesting a sound estimation of posterior distributions. The resulting molecular tree was split into two basal clades (X and Y), both supported with the highest posterior probabilities (Fig. 3). Both basal clades were further subdivided into two major clades (A, B, and C, D, respectively), all supported with high posterior probabilities. Some terminal clades were also highly supported, but only the division into the four major clades was used to calculate between and within group p-distances in our molecular analyses, as well as in LBGM analyses to study the evolution of shape and size. The p-distances between Baikal species (not shown) in the 18S alignment varied between 1 and 5%, while in the 16S alignment they varied between 1 and 15%. For the 18S the between group p-distances varied between 0.6 and 3.2%, while the within group distances varied between 0.4 and 1.7% (Supplementary Table S1). For the 16S the between group distances varied between 6.9 and 10.6%, while the within group distances were around 5% for all four major clades (Supplementary Table S2).

The trees reconstructed for the alignments of individual genes (not shown) were not well resolved. However, both were split into the same two basal clades (X and Y), supported by maximum posterior probabilities. While the four major clades were not recovered on the 18S tree, they were present on the 16S tree. The clades C and D received a high support (0.91 and 1, respectively), while the support for the clades A and B was around 0.7.

A parametric ANOVA (Table 1) showed significant differences between species in shape, and even more so in size. They were, respectively, over 46 times (estimated from the Goodall’s F critical value) and almost 470 times larger than individual variability, suggesting a significant allometry in the dataset. Directional asymmetry was large and statistically highly significant (p < 0.0001) for both shape (F above 55) and size (F above 92), prompting us to analyze separately the right valve (RV) and the left valve (LV). Sexual shape dimorphism was also significant (p < 0.001), but it was only 4.2 times larger than individual variability. However, it was much smaller than either directional asymmetry or the effect of species, so it was not necessary to separate males and females in our analyses. Sexual size dimorphism was non-significant (p = 0.6255).

**Table 1:** Shape and size variation of Baikal Candonidae inferred by a parametric ANOVA: *SS* sum of squares, *MS* mean squares, *df* degrees of freedom, *F* Goodall’s F critical value, *p* probability of finding a random value larger than the observed value, *PT* Pillai’s trace.

	Effects	SS	MS	df	F	p	PT	p
Shape	Species	1.49618363	0.0003696106	4048	46.53	< 0.0001	31.86	< 0.0001
	Sex	0.00306682	0.0000333350	92	4.20	< 0.0001	0.60	0.8836
	Individual	0.10012588	0.0000079440	12,604	1.00	0.4994	39.92	0.1375
	Directional asymmetry	0.04045329	0.0004397096	92	55.35	< 0.0001	0.90	< 0.0001
	Fluctuating asymmetry	0.13301226	0.0000079439	16,744	–	–	–	–
Size	Species	157,852,066.617706	3,587,546.968584	44	469.78	< 0.0001	–	–
	Sex	1826.982360	1826.982360	1	0.24	0.6255	–	–
	Individual	1,046,218.712071	7636.632935	137	1.46	0.0084	–	–
	Directional asymmetry	484,024.167354	484,024.167354	1	92.67	< 0.0001	–	–
	Fluctuating asymmetry	950,613.463865	5223.150900	182	–	–	–	–

Total allometry (calculated as a linear regression of Procrustes coordinates onto log transformed centroid size (CS)) in the RV species-averages dataset was significant (p = 0.0011) and accounted for almost 14% of total variability; while the evolutionary allometry (calculated as a regression of independent contrasts of shape onto independent contrasts of log transformed CS) was around 10% and was barely significant (p = 0.047). The pooled within-taxa regression was less than 1%, although the sample size for many species was probably too small for this analysis.

**Figure 4:**
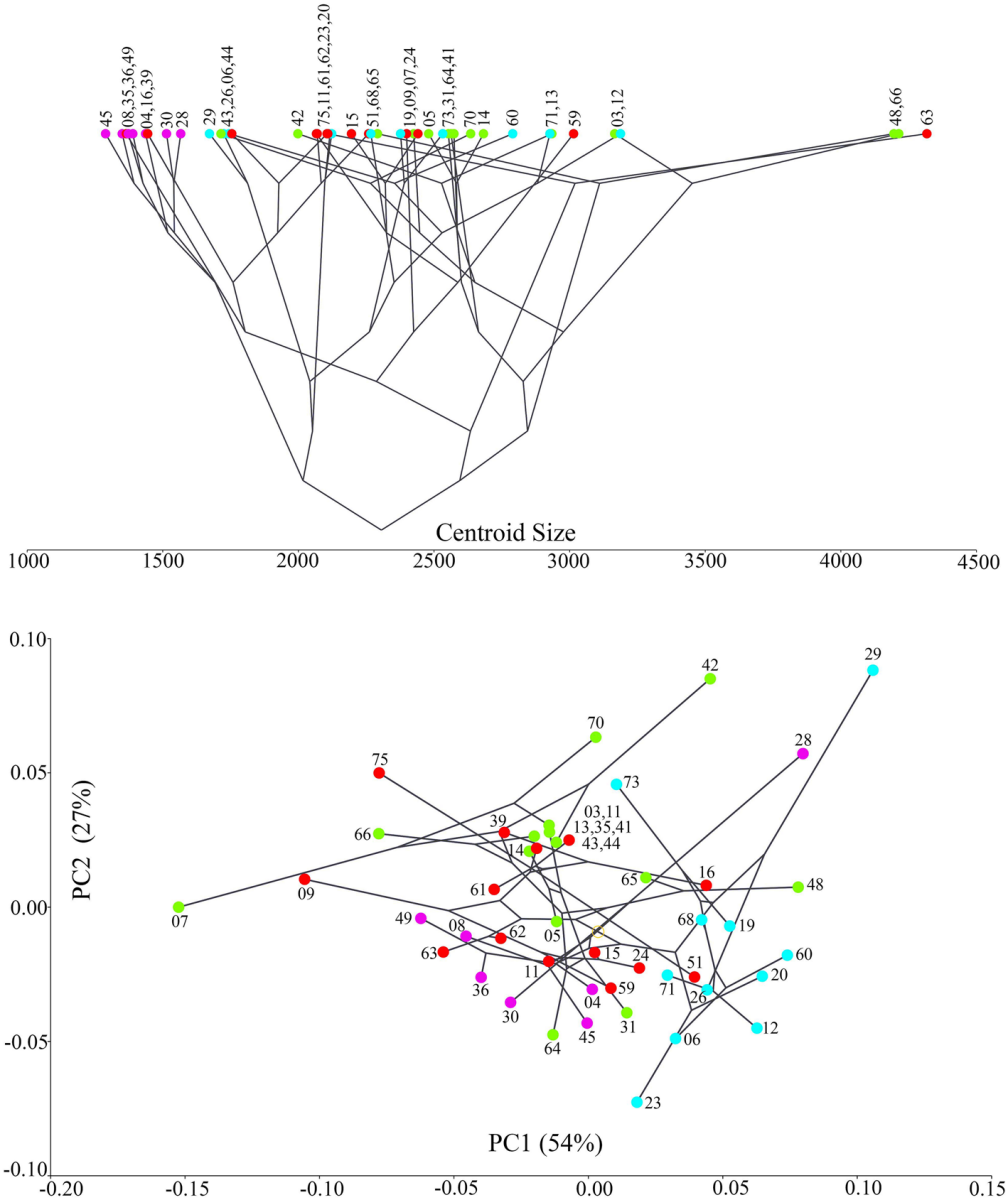
Projection of the Baikal Candonidae phylogeny (see Fig. 3) onto centroid size for each species mean value (top) and the first two principal component (PC) scores (bottom), using squared-change parsimony for the size-corrected right valve dataset. PCA analysis is based on the covariance matrix of regression residuals for the species-averages dataset. Numbers in brackets represent eigenvalues for the PCs, and numbers next to dots are species codes (for details see Supplementary Table S3). Color codes for major clades (**A**–**D**) as in Fig. 3 and in all Supplementary Figures.

**Figure 5:**
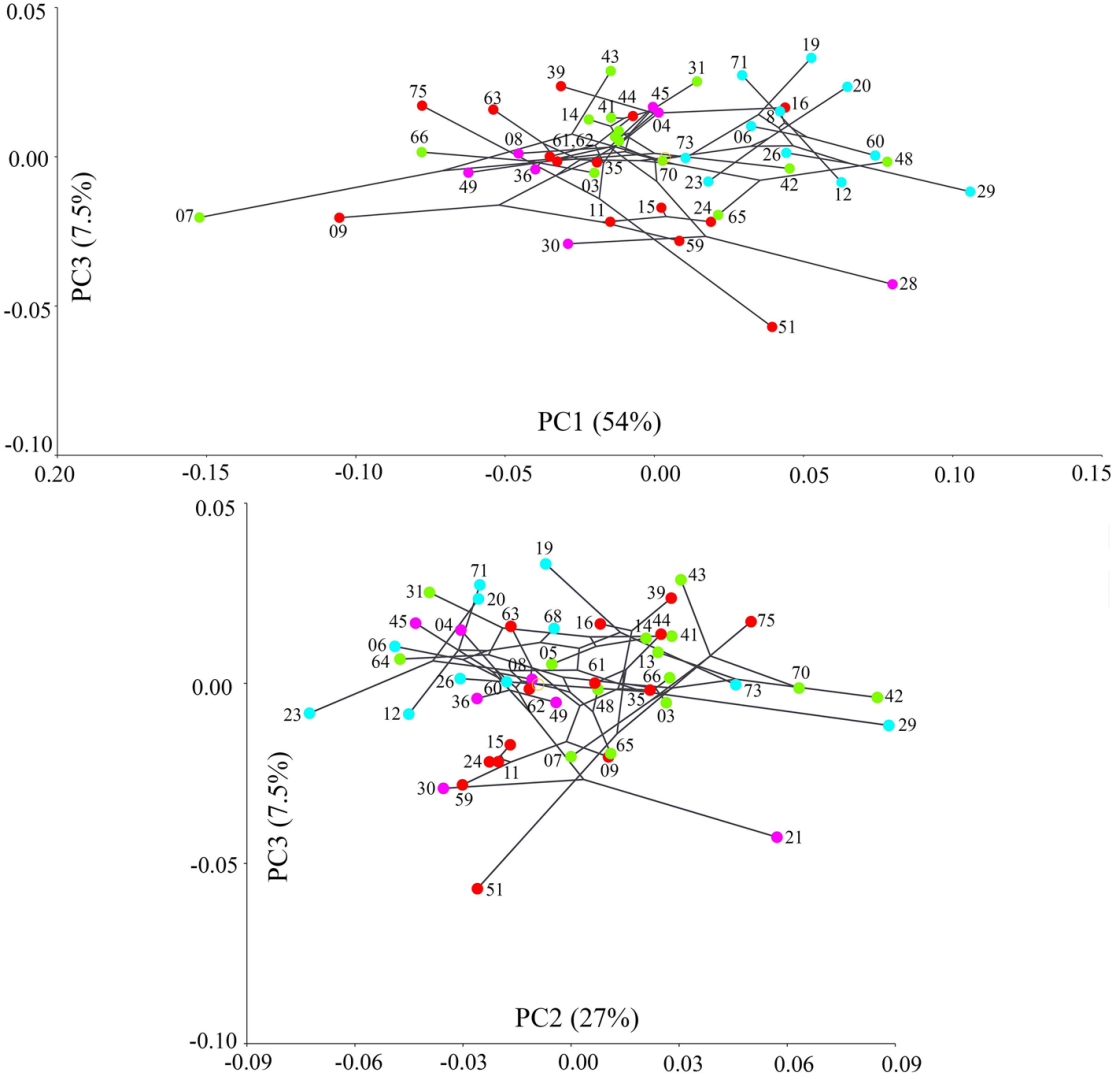
Projection of the Baikal Candonidae phylogeny (see Fig. 3) onto the first and third principal component (PC) scores (top) and the second and third PC scores (bottom), using squared-change parsimony for the size-corrected right valve dataset. Explanations as in Fig. 4.

**Figure 6:**
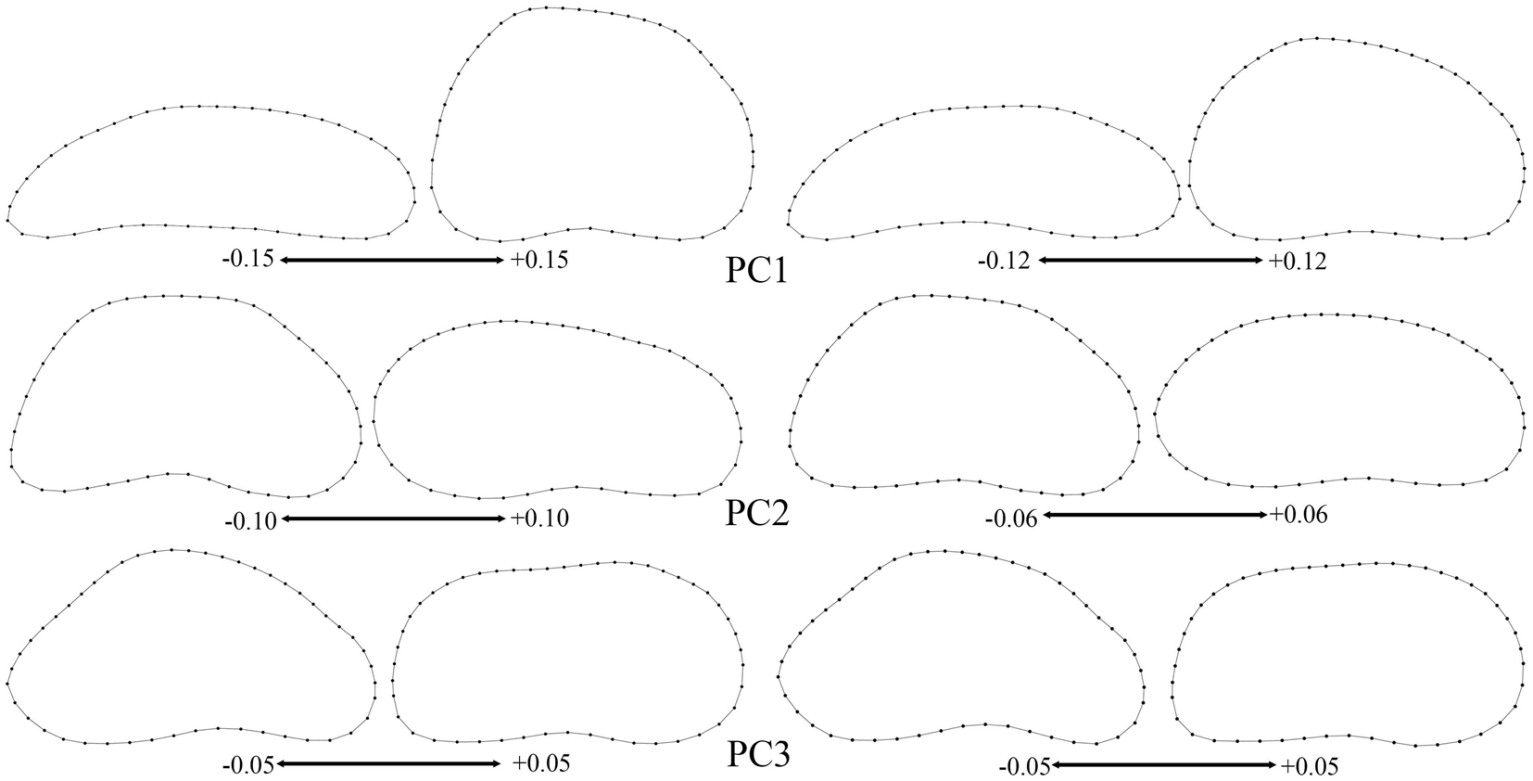
Patterns of the right valve shape changes associated with the first three principal components (PCs) of the size-corrected dataset (see Figs. 4, 5). Left: complete shape variations; right: phylogenetically independent contrasts. Scales show extremes of variability.

Principal Component Analysis (PCA) of the RV based on the covariance matrix of regression residuals of species-averages dataset revealed that slightly over 80% of variability could be explained by the first two eigenvectors (PCs), while the eigenvalue for PC3 was only 7.5% (Figs. 4, 5, Supplementary Figs. S1, S2). The clade C was completely in the positive part of PC1, while the clade D was mostly concentrated in the negative part of PC1 (with the exception of species 28). The clades A and B were scattered throughout the morphospace, although their centers were separated slightly by PC2. PC3 showed an almost complete overlap between all clades, but it did show less overlap between the clades A and B than PC1 or PC2. The shape changes associated with PC1 (Fig. 6) were in the general direction of elongation of the valve from very trapezoidal, influencing the width of the anterior and posterior margins. Those associated with PC2 were in the rounding of the posterior margin and in the slope of the dorsal margin, while those associated with PC3 were in the rounding of both anterior and posterior margins and in the change from triangular to rectangular shapes. None of the PCs described considerable changes in the ventral margin.

PCA of the RV based on the covariance matrix of independent contrasts produced very similar results to the previous analysis, in terms of the amount of variability carried by the first three PCs (Supplementary Fig. S2). The shape changes (Fig. 6) associated with PC1 and PC2 were slightly different in comparison to the shape changes associated with PCA of species-averages. Although PC1 described valve elongation, it was less pronounced, as was the sloppiness of the dorsal margin described by PC2. PC3 showed no difference between the two analyses. Shape changes associated with the evolutionary allometry (Supplementary Fig. S3) also described valve elongation, and were similar to the shape changes associated with PC2 of independent contrasts.

Plotting the phylogeny onto the shape tangent space by squared-change parsimony revealed that closely related taxa are not close to each other in morphospace and also that some distant taxa have similar shapes, resulting in long branches crisscrossing the plot. This was more pronounced when plotting the tree on PC1 and PC2 (Fig. 4), than on PC1 and PC3 or on PC2 and PC3 (Fig. 5), corresponding to the amount of variability associated with those PCs. The null hypothesis of no phylogenetic signal in the shape variation of the RV was rejected, because the permutation test was statistically significant (p = 0.0058). It was even more significant for size (p < 0.0001). With the exception of clade D, which was concentrated in the smaller end of size ranges, the other three clades showed a large variability. Sister species were either far apart in the shape tangent space (e.g., species 07 and 70; 16 and 39; 19 and 73, 23 and 20; 26 and 29; 28 and 30; 51 and 75; etc.), differed significantly in size (e.g. species 06 and 60; 44 and 61; 62 and 63; etc.), or both (e.g. species 03 and 66; 48 and 65; etc.) (see Fig. 4).

Shape changes at ancestral nodes of the phylogenetic tree (Fig. 3) indicated that the ancestral shape of all examined Baikal Candonidae can be defined as subtrapezoidal, and has stayed more or less unchanged in the ancestor of the basal clade Y and its two major clades (C and D). On the other hand, the ancestral shape of the basal clade X and its two major clades (A and B) has a slightly more rounded dorsal margin, resulting in a bean-like shape.

The LV showed even more allometry than the RV. In the species-averages dataset it accounted for almost 21% of total variability, while for independent contrasts it was 15.49%, and the latter was more statistically significant (p = 0.0006) than in the RV dataset. The results of the PCA based on the LV covariance matrix of regression residuals of species-averages was similar to the RV, with the first two PCs accounting for nearly 80% of total variability (Supplementary Figs. S4–S7). However, PC1 in the LV carried even more variability than in the RV (60.6% vs 54%), PC2 was lower (18.8% vs 27%), while PC3 was slightly higher (8.5% vs 7.5%). Shape changes associated with the first three PCs of species-averages, independent contrasts, and evolutionary allometry (Supplementary Fig. S8) were similar to those for the RV. Plotting the phylogeny onto the shape tangent space for the LV produced similar results to those for the RV, and the phylogenetic signal was significant (p = 0.0028). It was even more statistically significant for size (p < 0.0001). The distribution of species across the shape space and size ranges was similar to the RV, and the differences in size between sister species were even more pronounced (Supplementary Fig. S4).

## Discussion

The shell of LB Candonidae is characterized by a differentiation between closely related species in shape, size, or both, as well as by a convergent evolution. It mirrors a fast morphological diversification in a short period of time, as a consequence of adaptive radiation. This is congruent with previous studies on almost all other Baikal groups^46–49^. Baikal Candonidae, unlike many other animal groups, are currently distributed in all three basins and in all water depths. Previous studies found that most species were present in all three basins and had a wide bathymetric distribution^23^, although few were found below 400 m. Our sampling was limited, but we found no species in more than one basin or across several depths. In fact, most species were restricted in distribution to individual sampling localities. More so, closely related species were often found in different basins, different depths, or both. The major four phylogenetic clades were not basin or depth restricted, although two of them (C and D) were collected mostly from shallow waters (with only two species in each in waters deeper than 100 m). These two clades also have a deeper cladogenesis and are morphologically more conservative, most closely resembling their ancestors. Our data regarding basin and depth separation of sister species are highly congruent with another large LB ostracod group^15^. Studies on amphipods also show a limited distribution for most species, and those few that live throughout the lake have genetically isolated populations in different basins^50^.

In comparison to the concatenated dataset results, our phylogenetic trees based on individual markers had low branch supports and numerous polytomies. This is not unexpected for 18S, which is a complex gene, with a mixture of fast and slow evolving regions^51^. Most commonly, only the slow evolving regions are amplified, and they have low between species distances across animal taxa, regardless of the calculation method^52^. The 18S p-distances between the four major clades recovered in our concatenated dataset are similar to those found on the genus level in other ostracod groups^53,54^. The 16S has a much higher rate of evolution than 18S, and it is often used for species delineation in crustaceans^55,56^, sometimes even as a barcoding substitute in different animal groups^57–59^. One of the more complete studies on crustaceans^55^, which included calibrations with multiple geological events, suggested mutation rates for 16S between 0.38 and 0.9% per Ma. If those rates are applied to the LB Candonidae, their diversification started between 12 and 5.5 Ma. The 16S data for other Candonidae are unfortunately very limited. A recent study that applied a molecular clock to the 28S rRNA dataset, based on 38 species from around the world and calibrated with several fossil records, found very similar diversification age for the 10 LB species included^7^. There are only three other phylogenetic studies on ostracods comparable to ours, all on the family Cytherideidae in ancient lakes. One^16^ applied a molecular clock to the mtCOI dataset, based on 20 species of the genus *Cytherissa* Sars, 1925 from LB and calibrated with Wilke’s universal clock^60^, and estimated a diversification age between 8 and 5.38 Ma. Another study^15^ provided 16S data for the same genus, but did not use a molecular clock. Based on their row data, we calculated the diversification to fall between 20 and 8 Ma. Finally, a study on the Lake Tanganyika genus *Romecytheridea* Wouters, 1988 based on the 16S data^61^ calculated that species diversification started about 10 Ma. The fact that two unrelated LB ostracod groups showed similar diversification age, irrespective of the molecular markers used, gives us more confidence in our estimation.

Since previous studies^7^ used a variety of Candonidae, they were able to indicate that each of the two basal LB clades has their closest relatives in distinct Palearctic genera (*Fabaeformiscandona* Krstic, 1972 and *Candona*), and *Candona* seemed also to be sister to the entire LB clade. This is interesting because it can provide an insight into the origin of shell shape variations we detected with LBGM. Although *Fabaeformiscandona* and *Candona s.l.* are ripe for revision^26,62^, both genera display a variety of shell shapes.

The evolution of Candonidae started in the Middle Jurassic (~ 170 Ma), and the earliest fossil was recorded from Portugal^63^. It was well-adapted to the euryhaline waters of the Bajocian and survived subsequent changes of water salinity in the Upper Jurassic (the Oxfordian). Its shell was unusually ornamented (for Candonidae), and the general shape can be described as subtrapezoidal. This shape overwhelmingly predominates in the Cytheroidea, and it appears in many phylogenetically distant Candonidae, especially in those currently living in subterranean waters and in the fossils from the Late Miocene (the Pannon)^18^. The reconstructed ancestral shell shape for the LB Candonidae also has this general form, which persisted in the ancestor of the basal clade Y, and both of its major clades (C and D). These two clades also have the most concentrated morphospace occupancy, and are separated almost completely (save for one species) by PC1. The ancestor of the basal clade X has a more rounded dorsal margin, but still it maintains that subtrapezoidal appeal. Nevertheless, the morphological variability of its two major clades (A and B) is mirrored in their wider morphospace occupancy. In general, it seems that the shell of LB Candonidae mostly evolved along the morphological variation that describes elongated vs trapezoidal shapes, and the width of the anterior and posterior margins. PCA of the phylogenetically independent contrasts also shows that PC2 corresponds to the shape changes associated with size (evolutionary allometry).

In our PCA analyses, the first two PCs described around 80% of the total shape variation, indicating a strong integration. This was also found in many unrelated studies^2,4,64,65^. There is much evidence that high morphological integration can both promote and impair evolutionary potential of structures in different lineages^66,67^. A strong integration gives rise to a strong selection response, but along the paths of traits variation, which might limit morphological disparity and can lead to the evolution of extreme morphologies and convergences^68^. This seems to be the case not only with LB Candonidae, but Candonidae in general^20,25^. However, measures of morphological integration are influenced by a difference in morphometric representation (LBGM vs linear morphometrics) and the inclusion of size^69^. In addition, the number of PCs that describe shape variation also depends on LM choice. In recent studies of ostracods^29,31^ outline analyses were more structured than those based on internal LMs, yet the distribution of shape variation remained the same. Differences in integration between the outline and the internal LMs method in these studies were not a consequence of the number of LMs. The outline method is used more commonly in ostracods, because it is very difficult to find homologous structures on shells, especially when the surface is smooth, but it is possible in population studies of single species^29^.

Although there is a phylogenetic signal in our shape and size data, this might be overly influenced by a strong clustering of the clade D in both shape and size, and partly the clade C in shape. In both the LV and the RV datasets, size has a stronger phylogenetic signal than shape, and is statistically more significant. Evolutionary allometry contributes significantly to the shape variation, more so in the LV than the RV. In addition, PC1 for the LV had a slightly higher value. The reason for this might be that the LV in Candonidae always overlaps the RV on all free margins, and the valves are asymmetrical in both shape and size in all ostracods^20^. This was supported by our ANOVA results. When we plotted phylogeny onto the CS, the differences between species were more pronounced for the LV than the RV. Nevertheless, the shape variations related to the evolutionary allometry are very similar for both valves, and their extremes correspond to the shape variation along PC2. Asymmetry of the valves in ostracods is not limited to shape and size; recently it was found that they can also differ in the number of cuticular pores^29^. Ostracods appear to be unique among animals in the nature of their directional asymmetry^29^, and this subject certainly warrants further investigation.

So far the most impressive monographic work on LB Candonidae contains descriptions and redescriptions of 95 species and subspecies^23^. However, we were not able to match most of our specimens with them. It seems that at least some of these descriptions are collages of multiple species. Candonidae biodiversity assessment was not the aim of our study, but based on the unique shell shapes prevalent in our samples, we speculate that most of our examined species are new to science. This is in accordance with a recent study of the second largest ostracod group in LB, Cytherideidae, where the actual diversity might be double of what is currently known^15^; this study assessed biodiversity using two molecular markers (16S and 28S), and concluded that many of the collected species are actually cryptic. Declaring cryptic species seems to be a current trend in biodiversity related studies, and often it is a way to avoid thorough morphological investigations using LBGM^34,70,71^. Generally, biodiversity of all LB animals is understudied^12^, but it can be estimated properly with a combination of appropriate molecular markers, morphological studies, and taxonomic expertise^72,73^.

## Material and methods

### Sampling and taxonomy

Ostracods were collected during various expeditions from 2002 to 2017, on board four different research vessels (“Koptyg”, “Titov” (see Fig. 2A), “Papanin”, and “Vereschagin”), from a submersible (“Mir”), and by SCUBA diving. For this study we chose 28 sampling sites (Fig. 2), listed in the Supplementary Note (including sampling gear, sediment type, and depth). Details on expeditions, sampling, and sorting methods can be found elsewhere^74,75^.

Collected animals were dissected using Zeiss Axiostar-plus dissecting microscope. The valves were separated from the soft body, and the latter was transferred into lysis buffer for DNA extraction. Exoskeleton of the soft body was afterwards dissected on a glass slide in a drop of CMC-10 Mounting Medium (Masters Company, Inc.), for observation under Leica DM 2500 compound microscope, equipped with N-plan objectives. The valves were mounted on aluminum stubs, gold or platinum coated, and observed and photographed with a Hitachi S-4700 Scanning Electron Microscope.

Close to 80 species of Candonidae were recovered from the samples, but we were able to amplify both markers only for 46, which were used in further analyses (Supplementary Table S3). A wide variety of shell shapes was represented by those selected species (Fig. 1). To identify them, we used published keys, descriptions, and illustrations of both shell and soft body parts^23^. Unfortunately, less than a third of selected species could be identified, due to insufficient descriptions and presence of clearly new species. Therefore, all species were arbitrarily assigned to the genus *Candona* Baird, 1845 and given a unique species code (Supplementary Table S3) that was used throughout this paper (Figs. 1, 3, 4, 5; Supplementary Figs. S4, S5). Systematics follows a recently published revision^76^, where Candonidae were erected from the subfamily to the family level.

### DNA extraction and phylogeny

Lysis buffer was prepared according to the published protocols for nematods^77^. All PCR reactions were carried out in 25 µl volume, containing: 5 μl of DNA template, 2.5 μl of 10 × ExTaq Buffer, 0.25 μl of TaKaRa Ex Taq (5 units/μl), 2 μl of dNTP Mixture (2.5 mM each), 1 μl of each primer, and 13.25 μl of distilled water. Partial sequences of 18S rRNA and 16S rRNA were amplified using the PCR protocols and primers listed in Supplementary Table S4. The presence of DNA was verified with 1% agarose gel electrophoresis. After a treatment with LaboPass PCR Purification Kit (Cosmo Genetech), the PCR products were sequenced with ABI automated capillary sequencer (Macrogen, Seoul, South Korea), using the same sets of primers. Finch TV 1.4.0 (http://www.geospiza.com/Products/finchtv.shtml) was used to check for the quality of signal and sites with possible low resolution, which were corrected by comparing forward and reverse strands. BLAST algorithm^78^ was used to check the identity of obtained sequences. All sequences were deposited in GenBank (Supplementary Table S3).

Obtained sequences were aligned online^79^ with MAFFT v.7, and p-distances were calculated^80^ with MEGA 11. Sequences belonging to *Typhlocypris choi* Karanovic & Lee, 2012 (species code 01 in Fig. 3) and *Trapezicandona *sp*.* (species code 02) were chosen as outgroups (GenBank numbers for 18S: MF116214, MF116205; and for 16S: MF115629, MF115632, respectively). To improve alignments, indels were converted to a matrix of binary characters, using FastGap^81^. Alignments of 18S and 16S were concatenated, and the evolutionary model was tested using IQ-TREE Web Server^82^, applying Akaike information criterion^83^. Bayesian Inference, implemented in BEAST v2.5, was used to estimate phylogenetic relationships^84^. The analysis run three times for 10 million generations each, sampling every 1000 generations. Tracer^85^ was used to visualize the results of BEAST analyses, and FigTree v1.4.3 (available from http://github.com/rambaut/figtree) was used for tree visualization. Both markers were also analyzed separately, using the same methods.

### Morphometric data collection and analyses

A total of 386 valves, belonging to 202 individuals, and 46 species, were used for LBGM analyses (Supplementary Table S3). For 20 individuals one of the valves was damaged during dissection. A scale factor was set based on each SEM image magnification and 48 LMs were digitized in TpsDig 2.18, as the Cartesian (raw) coordinates^86^. Initially, two LMs were chosen based on their position, representing the most anterior (LM1) and the most posterior end of the valve (LM2), always with the same valve orientation, with ventral margin horizontal. They were used as anchoring points in defining the outline, consisting of 46 (28 dorsal and 18 ventral) equidistant pseudo LMs (to stress the fact that the points selected for the analysis do not correspond to specific anatomical locations). Outline points were converted to LMs using the sliding method, implemented in TpsUtil^87^.

All LBGM and statistical analyses were performed with algorithms implemented in MorphoJ 1.06d^88^. Cartesian coordinates were aligned using generalized Procrustes superimposition^89^. Outliers were checked manually for a possible landmark swap. To quantify the amounts of variation at different levels, we used a parametric ANOVA^90^, which is especially designed for the assessment of left–right asymmetry^91^. It was automatically performed for both size (univariate) and shape (multivariate), and we tested the effects of species, sex, variation among individuals, directional asymmetry (variation between valve sides), and fluctuating asymmetry (interaction of individual and side). Measurement error was not tested, because of the scale of differences between species. For all further analyses the average species shapes were used, and the original dataset was divided into the RV and the LV. Allometry was tested on three levels: as total allometry (by multivariate regression of shape onto log-transformed CS of the species averages); evolutionary allometry (by multivariate regression of independent contrasts of shape onto the independent contrasts of log-transformed CS); and total allometry accounting for the effect of species (by multivariate regression of shape onto log-transformed CS, pooled within species). Most analyses were performed on residuals of the size-corrected data^92^. To explore and visualize the shape variation in the datasets, we carried out the Principal Component Analysis (PCA) at two different levels: among the species averages and among independent contrasts (the latter corresponding to evolutionary changes). The phylogenetic signal in shape was tested, and the shape changes at different nodes of the phylogenetic tree were obtained by plotting the PCA scores onto the concatenated tree, using square-change parsimony and 10,000 random permutations^93^. The phylogenetic signal in size was studied by plotting CS onto the concatenated tree. All shape changes at the observed extremes of variability were visualized by wireframe graphs, constructed by linking neighboring LMs in MorphoJ.

## Supplementary Information

Supplementary Information.

## Data Availability

DNA sequences used in this study are available from GenBank; accession numbers are listed in the Supplementary Table S3.
