# Supplementary material for: Diversification of the shell shape and size in Baikal Candonidae ostracods inferred from molecular phylogeny
Source: Sci Rep. 2023 Feb 20;13:2950. doi: 10.1038/s41598-023-30003-5 (PMC9941104; doi:10.1038/s41598-023-30003-5)
Supplement: Supplementary file 1 — Supplementary Information. [file 41598_2023_30003_MOESM1_ESM.docx]

**Diversification of the shell shape and size in Baikal Candonidae ostracods in the light of molecular phylogeny**

Ivana Karanovic^1^, Huyen T. M. Pham ^1^ & Tanya Sitnikova^2^

^1^ Department of Life Science, Research Institute for Convergence of Basic Science, College of Natural Sciences, Hanyang University, Seoul, 04763, Republic of Korea. ^2^ Limnological Institute, Siberian Branch, Russian Academy of Sciences, Irkutsk, Russia. Correspondence and requests for material should be addressed to I.K. (email: ivana@hanyang.ac.kr)

SUPPLEMENTARY NOTE:

**List of sampling localities (numbers correspond to those on the map in Fig. 2):**

1. Frolikha hydrothermal seep, 55°31.305’N 109°46.666’E, 3 July 2017, depth 414 m; bottom sediment: aleurite, oxic layer 1 to 2 mm, woody detritus, copepod/diatom detritus, bacterial mats; sampling gear: box-corer; collectors: T. Sitnikova & T. Naumova.

2. Frolikha hydrothermal seep, 55°31.138’N 109°46.395’E, 5 July 2017, depth 435 m; bottom sediment: dark-brown aleurite, woody detritus, bacterial mats; sampling gear: box-corer; collectors: T. Sitnikova & T. Naumova.

3. Frolikha hydrothermal seep, approximately 55°31’N 109°46’E, 23 July 2010, depth 409 m; bottom sediment: grey aleurite, bacterial mats, woody/copepod detritus, mica plates; sampling gear: submersible “Mir-2”; collector: T. Sitnikova.

4. Frolikha hydrothermal seep, approximately 55°31’N 109°46’E, 25 July 2010, depth 433 m; bottom sediment: aleurite, bacterial mats; sampling gear: submersible “Mir-2”; collector: T. Sitnikova.

5. North-eastern Baikal, near Urbican Cape, 54°47.133’N 109°37.183’E, 18 September 2014, depth 10 m; bottom sediment: stones, pebbles, sand; sampling gear: dredge; collector: T. Sitnikova.

6. North-eastern Baikal, Davsha Bay, 54°21.600’N 109°28.31718’E, 18 September 2014, depth 10 m; bottom sediment: sand, detritus; sampling gear: dredge; collector: T. Sitnikova.

7. Soskovka Bay, 54°09.117’N 109°33.217’E, 17 July 2002, depth 5–6 m; bottom sediment: sand between boulders; sampling gear: SCUBA diving; collector: T. Sitnikova.

8. Academic Ridge, mud volcano, 53°49.06’N 107°76.00’E, 1 July 2017, depth 675 m; bottom sediment: aleurite, gas hydrate, sediments mixed and porous; sampling gear: box-corer; collectors: T. Sitnikova & T. Naumova.

9. Maloe More, Kharin-Irgi Bay, 53°03.056N 106°54.276'E, 18 August 2017, depth 20-24 m; bottom sediment: silt, sand, water plants; sampling gear: dredge; collector: T. Sitnikova.

10. Maloe More, Zagli Bay, 53°02.0465’N 106°56.3917’E, 6 October 2017, depth 13-15 m; bottom sediment: Nostoc and Chara; sampling gear: dredge; collector: T. Sitnikova.

11. Maloe More, Sakhyurta, 53°01.6985’N 106°54.3681’E, 4 October 2017, depth 40 m; bottom sediment: coarse sand; sampling gear: dredge; collector: T. Sitnikova.

12. Maloe More strait, Small Olkhon Gate, 52°59.775’N, 106°55.235’E, 18 August 2017, depth 8 m; bottom sediment: sand, detritus; sampling gear: Peterson bottom grab; collector: T. Sitnikova.

13. Methane seep St-Petersburg, 52°52.371’N 107°09.307’E, 9 July 2017, depth 1479 m; bottom sediment: anoxic aleurite, grey sand, mica plates; sampling gear: box-corer; collectors: T. Sitnikova & T. Naumova.

14. Anga Bay, 52°47.237’N 106°36.205’E, 4 October 2017, depth 102-116 m; bottom sediment: mud; sampling gear: dredge; collector: T. Sitnikova.

15. Birhin Bay, 52°43.696’N 106°33.1237’E, 6 October 2017, depth 24-46-16 m; bottom sediment: fine sand and *Chara*; sampling gear: dredge; collector: T. Sitnikova.

16. Zelenovskaya oil-methane seep, 52°38.4444’N 107°21.6765’E, 3 July 2012, depth 275 m; bottom sediment: oxic aleurire from 2 to 3 cm, oil spots, bacterial mats, diatom detritus, smell of sulfur-hydrogen; sampling gear: box-corer; collectors: T. Sitnikova & T. Naumova.

17. Mud volcano K-2 (Kukui-2), 52°35.4650’N, 106°46.2820’E, 2 July 2013, depth 930 m; bottom sediment: aleurite, oxic layer about 5cm, small manganese iron crusts; sampling gear: box-corer; collectors: T. Sitnikova & T. Naumova.

18. Mud volcano K-9 (Kukui-9), 52°35.2290’N, 106°43.7020’E, 6 July 2013, depth 792 m; bottom sediment: oxic aleurite from 1 to 2 mm; sampling gear: box-corer; collectors: T. Sitnikova & T. Naumova.

19. Peschanaya Bay (see Fig. 2B), 52°10.4513’N 105°48.5667’E, 10 July 2012, depth 850 m; bottom sediment: oxic aleurite about 1 cm, diatom detritus, and fine quartz sand; sampling gear: box-corer; collectors: T. Sitnikova & T. Naumova.

20. Posolskaya Bank methane seep, 52°04.9382’N 105°49.7654’E, 8 July 2012, reference site, depth 183 m; bottom sediment: oxic aleurite from 1 to 2 cm, without iron-manganese crusts, without bacterial mats, hydrotroilite particles and copepod detritus; sampling gear: box-corer; collectors: T. Sitnikova & T. Naumova.

21. Posolskaya Bank methane seep, 52°02.2242N 105°50.6950’E, 6 July, 2012, depth 453 m; bottom sediment: oxic aleurite from 0.5 to 5 cm, with copepod/diatom detritus, without bacterial mats, gas-hydrate bedding deeper than 70 cm inside sediments; sampling gear: box-corer; collectors: T. Sitnikova & T. Naumova.

22. Posolskaya Bank, 52°02.1401'N 105 50.5852'E, 19 June 2010, depth 503 m; bottom sediment: aleurite, oxic layer ~ 0.5 cm, black silt, with bacterial mats; sampling gear: bottom grab “Okean-3”; collectors: T. Sitnikova & T. Naumova.

23. Bolshoye Goloustnoye, 52°01.443’N 105°23.930’E, 3 October 2017, methane seep, depth 117-125 m; bottom sediment: rocks, sand; sampling gear: dredge; collector: T. Sitnikova.

24.Bolshoye Goloustnoye, 52°01.249N 105°23.470’E, 24 August 2017, depth 5–7.5 m; bottom sediment: sand, silt, water plants, bacterial mats; sampling gear: dredge; collector: T. Sitnikova.

25. Bolshoye Goloustnoye, 51°59.6514N 105°21.5033’E, methane seep, depth 270 m, 17 June 2010; bottom sediment: aleurite, oxic layer around 1 cm, anoxic silt; sampling gear: box-corer; collector: T. Sitnikova.

26. Bolshoye Goloustnoye, 51°58.9837’N 105°21.3334’E, methane seep, depth 365 m, 17 June 2010; bottom sediment: aleurite, oxic layer 0.5 cm, grey silt with grey sand, bacterial mats, smell of hydrogen sulfide; sampling gear: box-corer; collector: T. Sitnikova.

27. Bolshiye Koty (see Fig. 2C), Zhilische, near Limnological Institute, 51°54.15’N 105°03.50’E depth 0.5 m, 1 July 2011; bottom sediment: stones and sand; sampling gear: hand net; collector: A. Poberezhnaya.

28. Bolshiye Koty (see Fig. 2C), Zhilische, near Limnological Institute, 51°54.15’N 105°03.50’E, depth 4 m, 1 July 2011; bottom sediment: sand (see Fig. 2D); sampling gear: SCUBA; collector: T. Sitnikova.

SUPPLEMENTARY FIGURES:


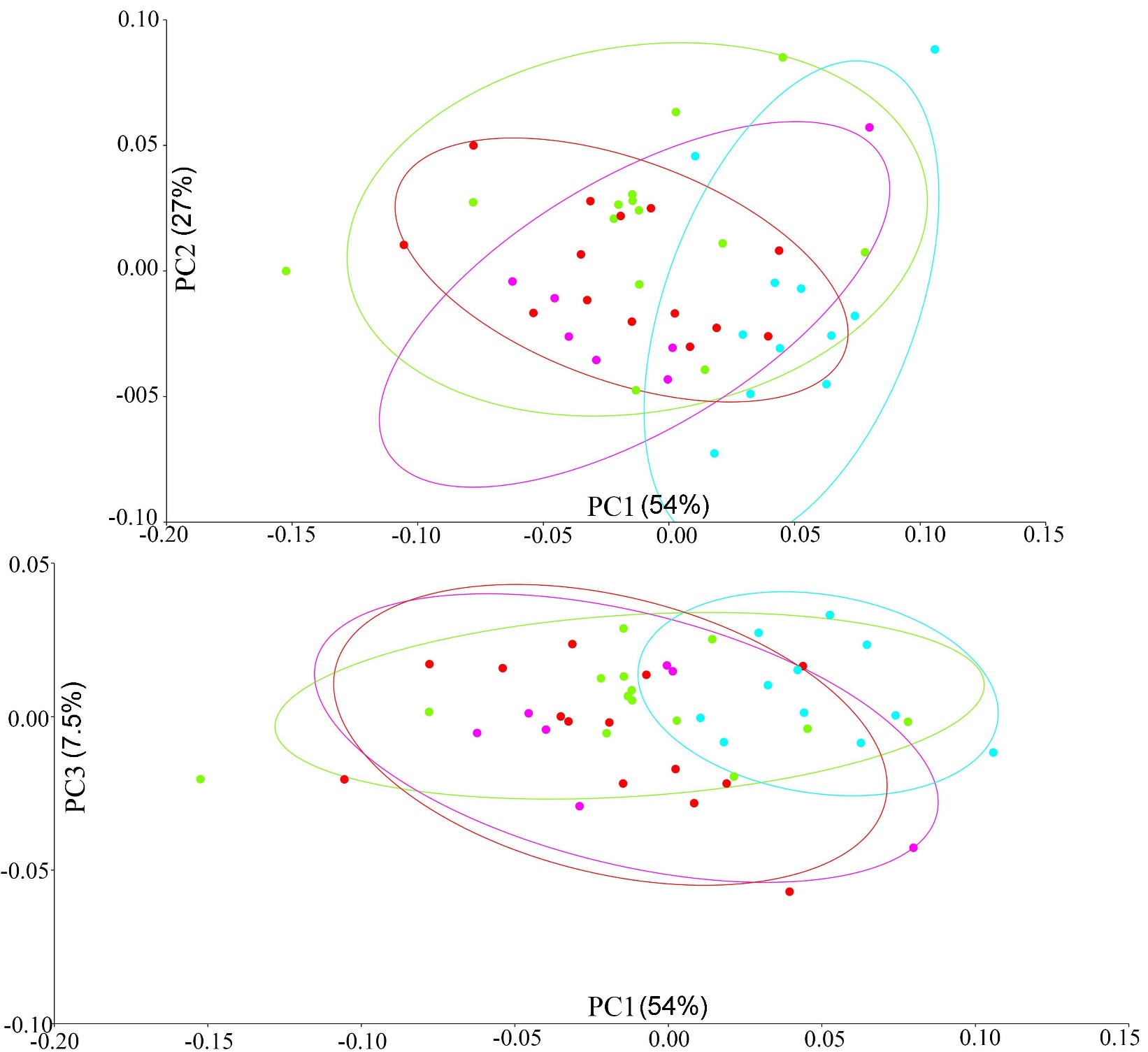


**Supplementary Figure S1.** Graphical visualization of the Principal Component Analysis for the right valve, averaged by species and size-corrected. Scatter plots show morphospace occupancy of the four major clades (A-D; see Fig. 3) along the first two principal components (PCs) (top), and first and third PCs (bottom). Ellipses represent 95% confidence intervals.


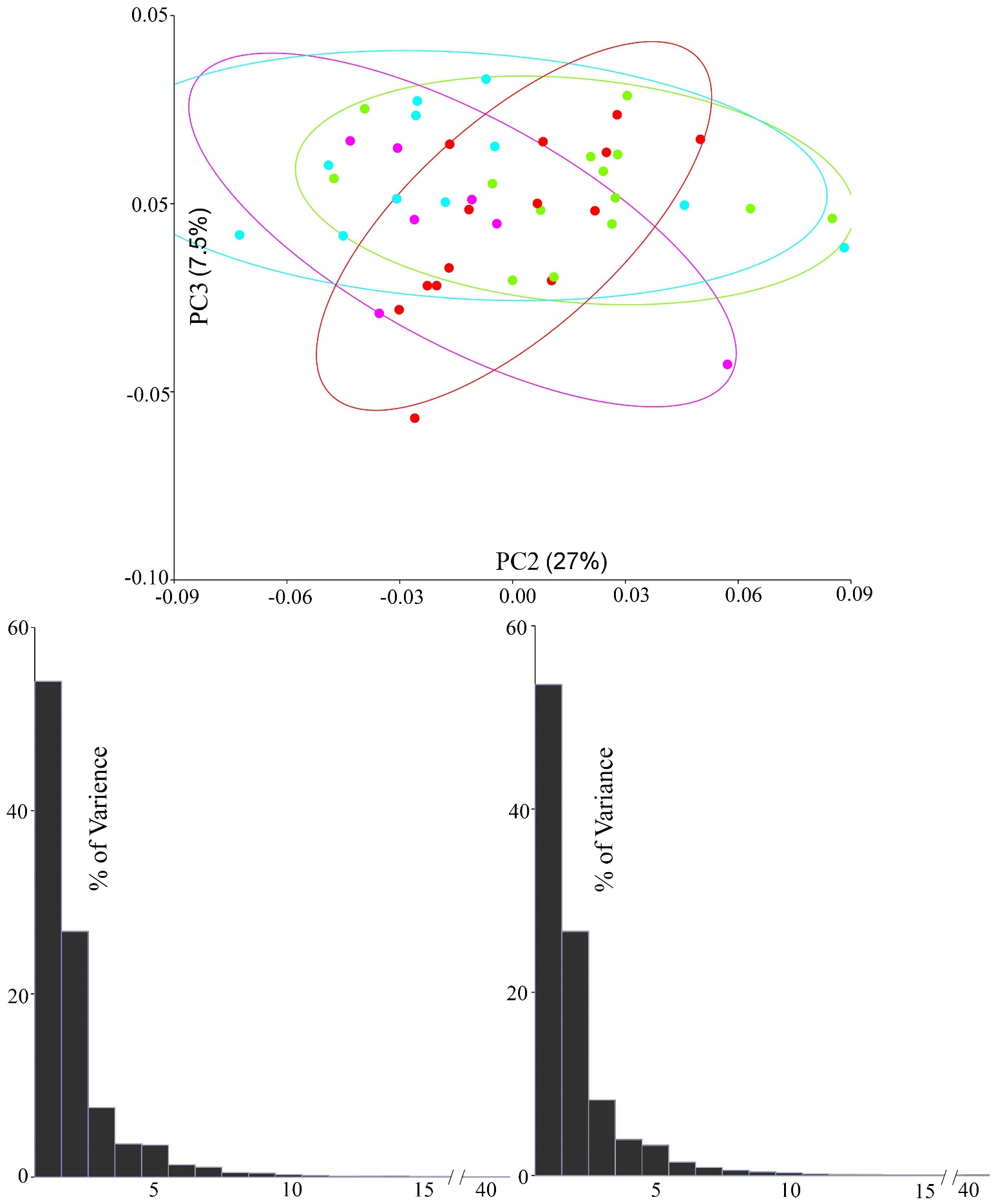


**Supplementary Figure S2.** Graphical visualization of the Principal Component Analysis for the right valve, averaged by species and size-corrected. Scatter plot shows morphospace occupancy of the four major clades (A-D; see Fig. 3) along the second and third principal components (PCs) (top); graphs (bottom) show percentages of the total variation for each PC in the size-corrected dataset (left) and for independent contrasts (right).


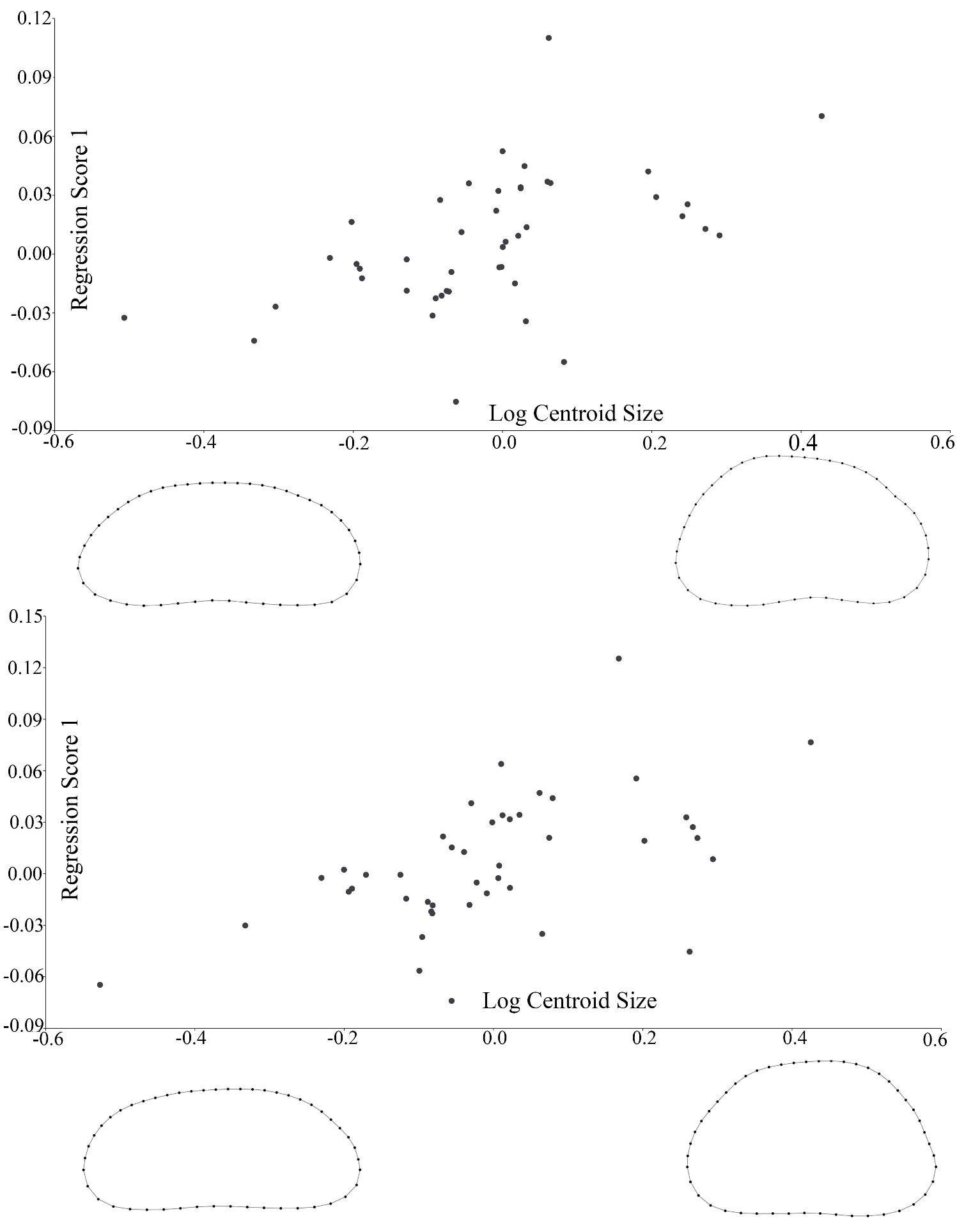


**Supplementary Figure S3.** Graphical visualization of evolutionary allometry, measured as a regression of independent contrasts of shape onto independent contrasts of log centroid size, for the right valve (top) and the left valve (bottom), and associated shape changes.


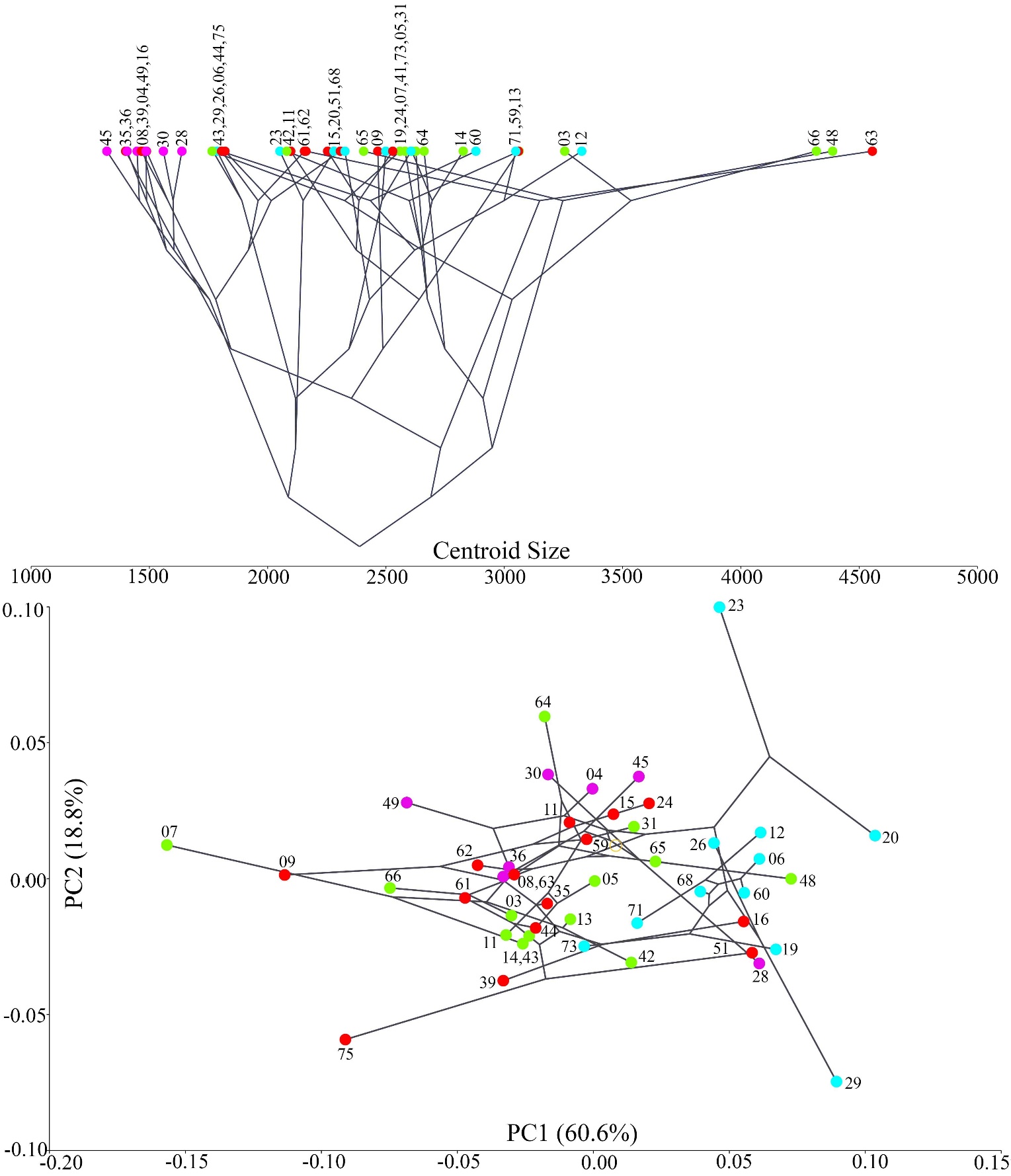


**Supplementary Figure S4.** Projection of the Baikal Candonidae phylogeny (see Fig. 3) onto centroid size for each species mean value (top) and the first two principal component (PC) scores (bottom), using squared-change parsimony for the size-corrected left valve dataset. Principal Component Analysis is based on the covariance matrix of regression residuals for the species-averages dataset. Numbers in brackets represent eigenvalues for the PCs, and numbers next to dots are species codes (Supplementary Table S3). Color codes for major clades as in Fig. 3.


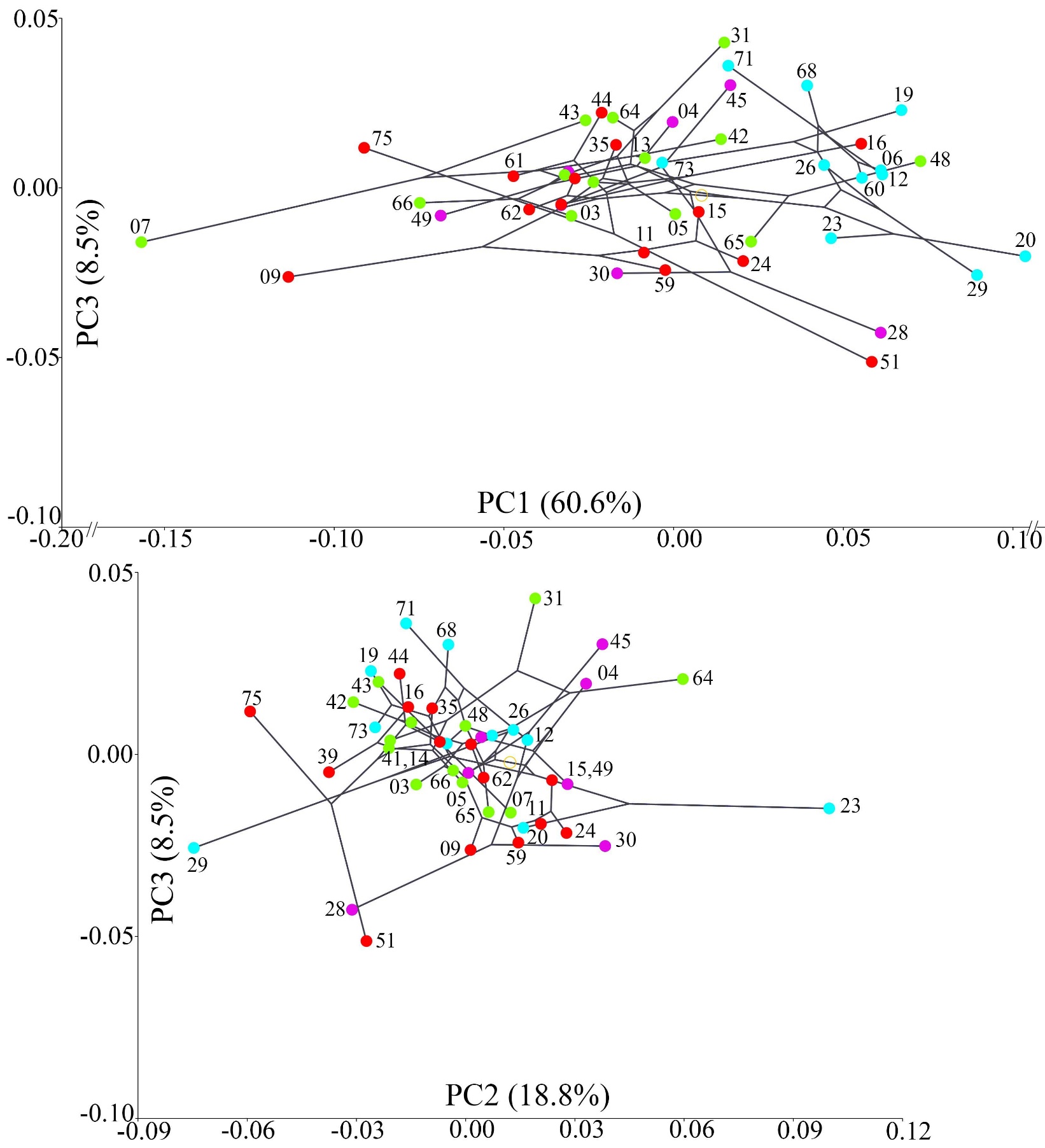


**Supplementary Figure S5.** Projection of the Baikal Candonidae phylogeny (see Fig. 3) onto the first and third principal component (PC) scores (top) and the second and third PC scores (bottom), using squared-change parsimony for the size-corrected left valve dataset. Explanations as in Supplementary Fig. S4.


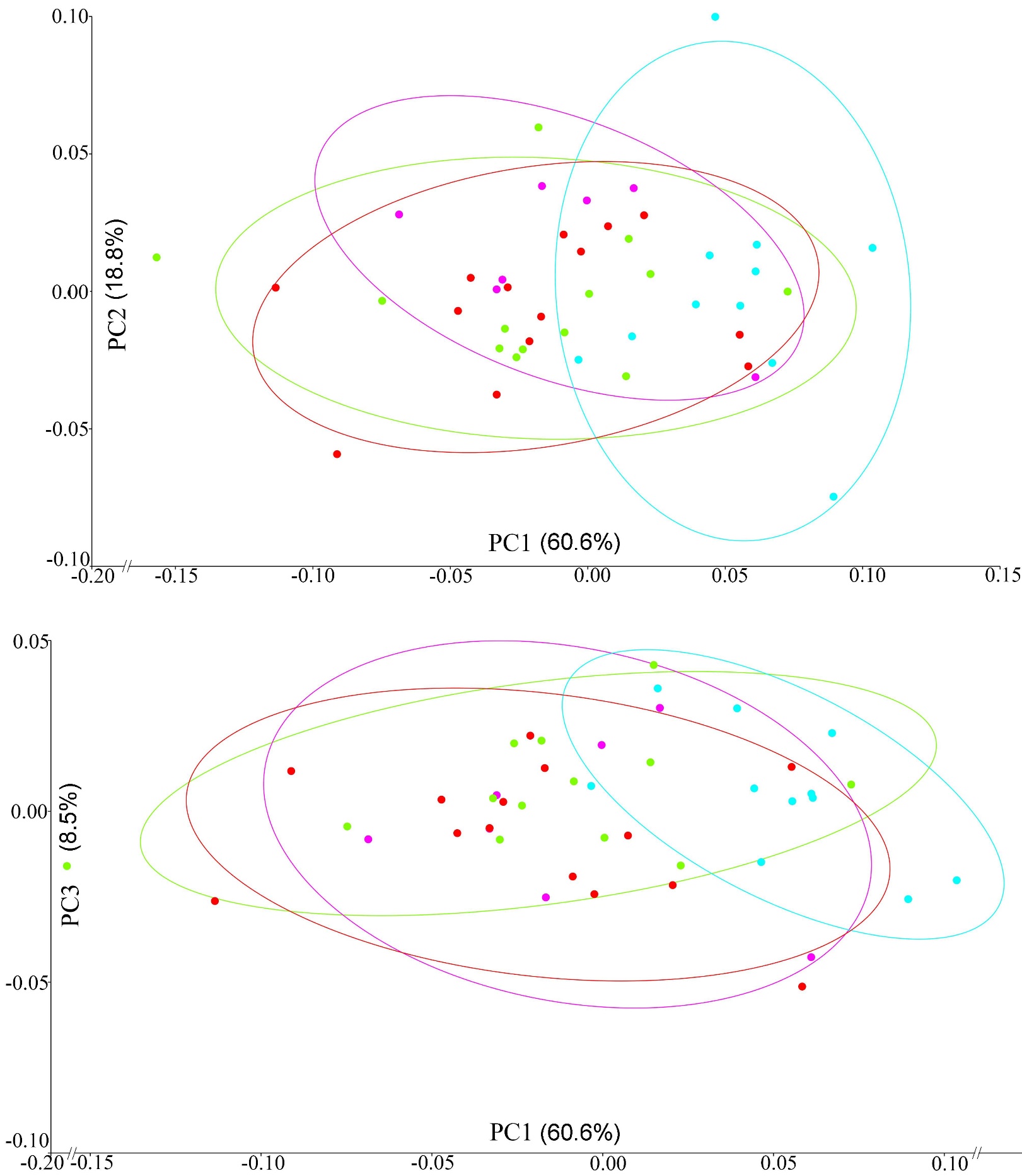


**Supplementary Figure S6.** Graphical visualization of the Principal Component Analysis for the left valve, averaged by species and size-corrected. Scatter plots show morphospace occupancy of the four major clades (A-D; see Fig. 3) along the first two principal components (PCs) (top), and first and third PCs (bottom). Ellipses represent 95% confidence intervals.


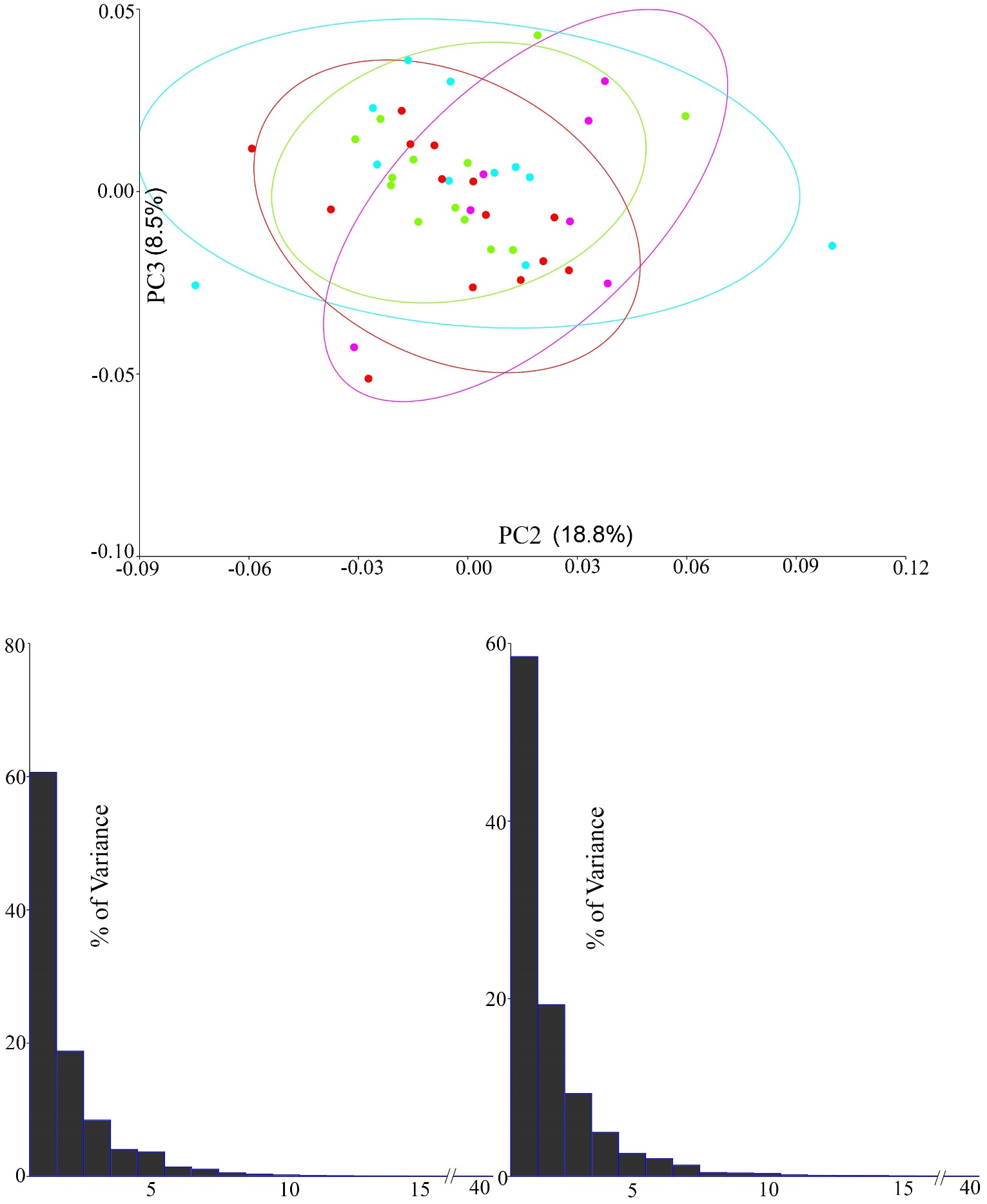


**Supplementary Figure S7.** Graphical visualization of the Principal Component Analysis for the left valve, averaged by species and size-corrected. Scatter plot shows morphospace occupancy of the four major clades along the second and third principal components (PCs) (top); graphs (bottom) show percentages of the total variation for each PC in the size-corrected dataset (left) and for independent contrasts (right).


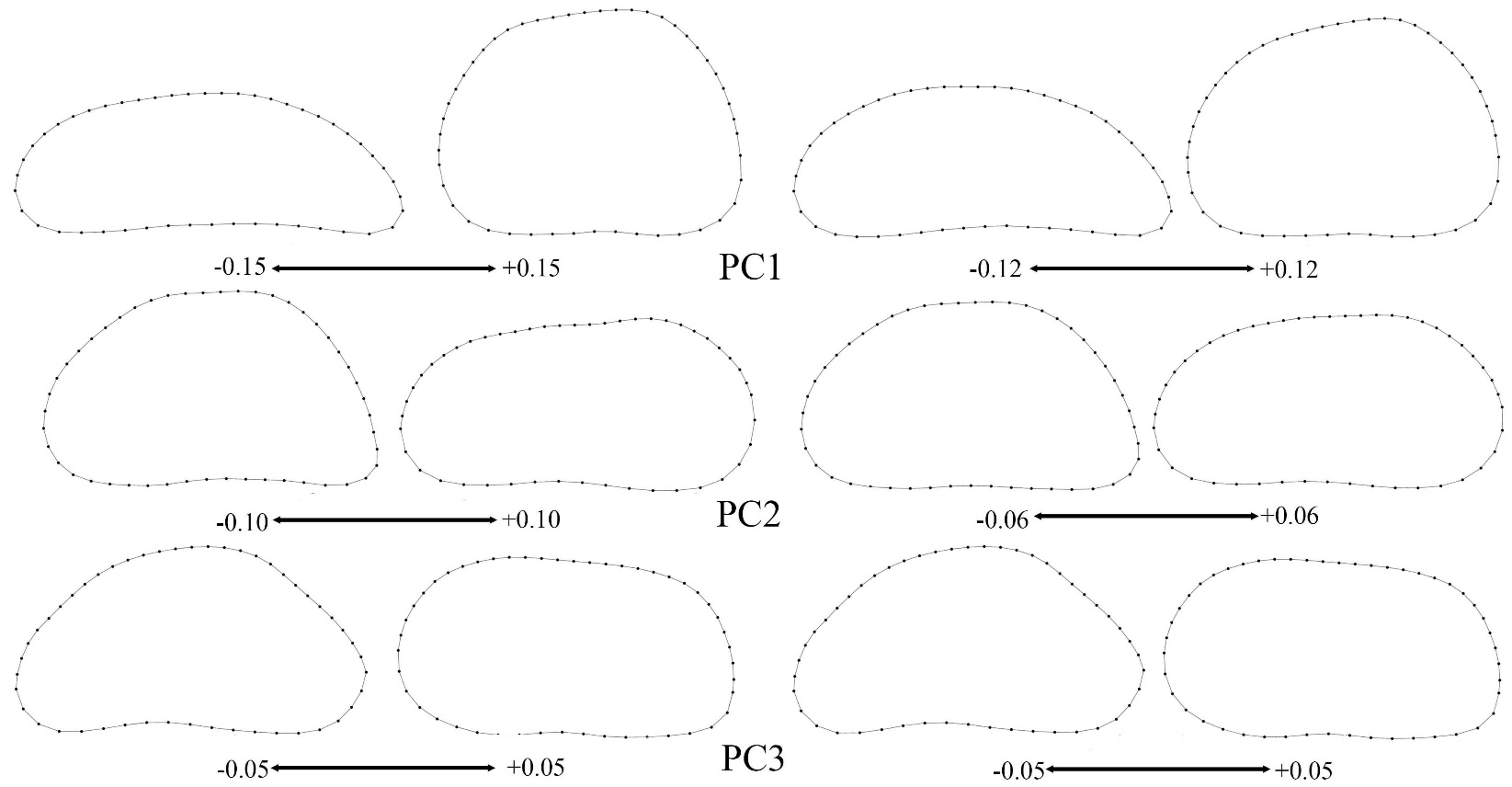


**Supplementary Figure S8.** Patterns of the left valve shape changes associated with the first three principal components (PCs) of the size-corrected dataset (see Supplementary Figs. S4 & S5). Left: complete shape variations; right: phylogenetically independent contrasts. Scales show extremes of variability.

SUPPLEMENTARY TABLES:

|  | **A** | **B** | **C** | **D** | **O** |
| --- | --- | --- | --- | --- | --- |
| A | 0.006 |  |  |  |  |
| B | 0.006 | 0.004 |  |  |  |
| C | 0.031 | 0.032 | 0.017 |  |  |
| D | 0.031 | 0.032 | 0.016 | 0.014 |  |
| O | 0.038 | 0.037 | 0.059 | 0.059 | 0.054 |

**Supplementary Table S1.** Within and between group p-distances for the partial 18S rRNA, calculated with 1000 bootstrap replications for four major clades (A-D; see Fig. 3) and outgroup taxa (O).

|  | **A** | **B** | **C** | **D** | **O** |
| --- | --- | --- | --- | --- | --- |
| A | 0.044 |  |  |  |  |
| B | 0.069 | 0.046 |  |  |  |
| C | 0.099 | 0.103 | 0.053 |  |  |
| D | 0.097 | 0.106 | 0.069 | 0.045 |  |
| O | 0.161 | 0.163 | 0.175 | 0.169 | 0.190 |

**Supplementary Table S2.** Within and between group p-distances for the partial 16S rRNA, calculated with 1000 bootstrap replications for four major clades (A-D; see Fig. 3) and outgroup taxa (O).

| **Species**  **code** | **Basin** | **Locality** | **Depth (m)** | **Male (84)** | | **Female (118)** | | **GenBank numbers** | |
| --- | --- | --- | --- | --- | --- | --- | --- | --- | --- |
|  |  |  |  | **RV** | **LV** | **RV** | **LV** | **18S rRNA** | **16S rRNA** |
| 3 | C | 14 | 102-116 | 5 | 5 | 4 | 4 | MW332408 | MW327084 |
| 4 | C | 14 | 102-116 | 4 | 4 | 8 | 8 | MW332418 | MW327095 |
| 5 | C | 14 | 102-116 | 2 | 2 | 3 | 4 | MW332433 | MW327112 |
| 6 | C | 10 | 13-15 | 2 | 2 | 6 | 6 | MW332453 | MW327150 |
| 7 | C | 10, 11 | 13-40 | 2 | 2 | 1 | 1 | MW332444 | MW327132 |
| 8 | C | 10 | 13-15 | 3 | 1 | 0 | 0 | MW332389 | MW327133 |
| 9 | S | 23 | 117-125 | 3 | 3 | 4 | 4 | MW332445 | MW327134 |
| 11 | C | 11 | 40 | 2 | 3 | 2 | 2 | MW332391 | MW327055 |
| 12 | C | 9, 15 | 20-46 | 1 | 1 | 2 | 2 | MW332391 | MW327055 |
| 13 | C | 13 | 1479 | 3 | 4 | 4 | 4 | MW332393 | MW327058 |
| 14 | N | 1 | 414 | 4 | 4 | 1 | 1 | MW332394 | MW327062 |
| 15 | N | 1 | 9-15 | 2 | 2 | 1 | 1 | MW332395 | MW327063 |
| 16 | C | 12 | 276 | 7 | 7 | 1 | 1 | MW332396 | MW327064 |
| 19 | S | 9 | 20-24 | 0 | 0 | 9 | 8 | MW332450 | MW327141 |
| 20 | S | 9 | 20-24 | 1 | 1 | 0 | 0 | MW332386 | MW327050 |
| 23 | N | 2 | 435 | 0 | 0 | 2 | 2 | MW332387 | MW327051 |
| 24 | N | 8 | 675 | 2 | 3 | 3 | 3 | MW332404 | MW327073 |
| 26 | S | 27 | 0.5 | 5 | 5 | 5 | 6 | MW332405 | MW327077 |
| 28 | S | 27 | 0.5 | 0 | 0 | 6 | 6 | MW332406 | MW327081 |
| 29 | S | 27 | 0.5 | 0 | 0 | 4 | 5 | MW332407 | MW327083 |
| 30 | S | 28 | 4 | 3 | 2 | 5 | 5 | MW332409 | MW327086 |
| 31 | N | 6 | 10 | 0 | 0 | 3 | 4 | MW332451 | MW327145 |
| 35 | N | 5 | 10 | 0 | 0 | 1 | 2 | MW332412 | MW327089 |
| 36 | N | 5 | 10 | 0 | 0 | 2 | 2 | MW332414 | MW327092 |
| 39 | N | 5 | 10 | 1 | 1 | 0 | 0 | MW332417 | MW327094 |
| 41 | C | 16, 18 | 275-792 | 6 | 6 | 6 | 6 | MW332420 | MW327099 |
| 42 | S | 19 | 850 | 3 | 2 | 0 | 1 | MW332423 | MW327102 |
| 43 | S | 21 | 453 | 1 | 1 | 1 | 1 | MW332425 | MW327104 |
| 44 | C | 16 | 275 | 2 | 2 | 2 | 2 | MW332426 | MW327105 |
| 45 | C | 16 | 275 | 0 | 0 | 1 | 1 | MW332427 | MW327107 |
| 48 | S | 20 | 183 | 0 | 0 | 2 | 2 | MW332430 | MW327110 |
| 49 | S | 20 | 183 | 1 | 0 | 1 | 1 | MW332431 | MW327111 |
| 51 | S | 27 | 0.5 | 1 | 1 | 0 | 0 | MW332435 | MW327115 |
| 59 | N | 7 | 5-6 | 5 | 5 | 5 | 4 | MW332436 | MW327121 |
| 60 | N | 7 | 5-6 | 2 | 3 | 0 | 0 | MW332455 | MW327152 |
| 61 | S | 26 | 365 | 2 | 2 | 1 | 1 | MW332437 | MW327122 |
| 62 | S | 26 | 365 | 1 | 1 | 0 | 0 | MW332438 | MW327125 |
| 63 | S | 25 | 270 | 0 | 0 | 1 | 1 | MW332439 | MW327126 |
| 64 | S | 22 | 503 | 0 | 0 | 1 | 1 | MW332388 | MW327053 |
| 65 | N | 3, 4 | 409-433 | 3 | 3 | 3 | 3 | MW332440 | MW327129 |
| 66 | N | 4 | 433 | 0 | 0 | 2 | 2 | MW332443 | MW327130 |
| 68 | S | 24 | 5-7.5 | 0 | 0 | 4 | 4 | MW332454 | MW327149 |
| 70 | C | 16 | 275 | 1 | 0 | 0 | 0 | MW332392 | MW327060 |
| 71 | N | 6 | 10 | 0 | 0 | 3 | 3 | MW332452 | MW327143 |
| 73 | C | 16 | 275 | 1 | 1 | 0 | 0 | MW332422 | MW327098 |
| 75 | C | 17 | 930 | 0 | 0 | 1 | 1 | MW332403 | MW327072 |
| Total | | | | 81 | 79 | 111 | 115 |  | |

**Supplementary Table S3.** Number of observations for the LBGM analyses, sampling localities, and GenBank numbers for sequences used in molecular analyses. Observations are listed per species, sex, and valves. Sampling locality numbers correspond to those in Fig. 2. Lake Baikal basins: C, central; N, north; S, south.

|  | **Primer sequences (5'-3')** | **PCR settings** |
| --- | --- | --- |
| 16S | F1-ATY AAA GAT AAG RCC TGC TCA ATG | 94°C 300s;  40 x [94°C 30s, 50°C 30s, 72°C 60s];  72°C 600s |
|  | F2-AAT AAT TTG CCT TTT AAT TGG GGG |  |
|  | R1-TTA ATT CAA CAT CGA GGT CGC |  |
|  | R2-ATT TYA AAA GTC GAA CAG ACT TAC |  |
| 18S | F1-TGA CGA AAA ATA ACA ATA CGG GAC | 94°C 300s;  40 x [94°C 30s, 48°C 60s, 72°C 60s];  72°C 600s |
|  | R1-GGCT AAA GTC TCG TTC GTT ATC |  |
|  | F2-GGG ACT CAT CCG AGG CCC CG |  |
|  | R2-GTC TGG GCC TGG TGA GGT TTC CCG |  |

**Supplementary Table S4.** Primers and PCR settings used for DNA amplification.
